# Editorial: Regulatory networks in genome stability pathways

**DOI:** 10.3389/fgene.2023.1171136

**Published:** 2023-03-14

**Authors:** Nicholas W. Ashton, Aishwarya Prakash, Tatiana N. Moiseeva

**Affiliations:** ^1^ National Institute of Child Health and Human Development, National Institutes of Health, Bethesda, MD, United States; ^2^ Mitchell Cancer Institute, University of South Alabama, Mobile, AL, United States; ^3^ Department of Chemistry and Biotechnology, Tallinn University of Technology, Tallinn, Estonia

**Keywords:** DNA repair signaling, post translational modifications, tumorigenesis, DNA replication, genome stability

Genetic alterations are major drivers of cancer initiation and progression. Although only a small portion of DNA mutations will confer cells with a selective growth or survival advantage, those that do can allow cells to overcome replication, cell death, and immunological limits. This, in turn, can allow cancers to develop, proliferate, and metastasize throughout the body (Hanahan and Weinberg, 2011). To prevent this scenario, cells use a wide range of genome stability maintenance mechanisms to ensure the DNA is protected, faithfully replicated, and repaired when damaged. These processes are controlled by a large network of regulatory pathways, that coordinate the expression, interactions and post-translational signaling required to ensure their appropriate use (Sirbu and Cortez, 2013; Dantuma and van Attikum, 2016). In this Research Topic, we aimed to publish articles that highlight the important roles of these networks and provide insight into how their disruption can contribute to carcinogenesis. The publications in this Research Topic cover various aspects of these networks–from specific DNA repair pathways to gene expression and mutation profiling–paving the way for potential future approaches to cancer diagnostics and treatment.

Two of the articles in this Research Topic provide specific examples of how DNA repair pathways can be controlled by regulatory proteins. In the study by Daniels et al. the authors demonstrate a novel means through which the DNA repair protein MLH1 is regulated by the ABL1 tyrosine-protein kinase. MLH1 is a core component of the mismatch repair pathway and is required for the excision and subsequent replacement of mispaired bases (Goellner, 2020). In their work, the authors demonstrate that MLH1 is phosphorylated by the ABL1 kinase and suggest that this modification helps to stabilize MLH1 by preventing its lysosomal degradation. This work thereby illustrates a new regulatory mechanism mediated by a post-translation modification. In contrast to this study, Payliss et al. review how SLX4 can coordinate the functions of numerous DNA nucleases *via* protein-protein interactions. SLX4 and its near-constitutive binding partner, SLX1, together function as a structure-specific endonuclease that cleaves a wide range of branched DNA molecules (Fricke and Brill, 2003). Here, the authors discuss how SLX4 acts as a protein-binding scaffold, allowing the SLX1-SLX4 dimer to form distinct complexes with numerous other proteins and thereby regulate a range of genome stability processes.

While the publications above describe specific mechanisms of regulation in the DNA repair pathways, other articles in the Research Topic sought to describe how the mutation and deregulation of genome stability mechanisms can contribute to carcinogenesis. Two articles did so by studying the pathogenicity of cancer-driving mutations in BRCA proteins. BRCA1 and BRCA2 function in the homologous recombination DNA repair pathway and are essential for coordinating the recruitment of other repair proteins (Prakash et al., 2015). In the publication by Doraczynska-Kowalik et al. the authors used genetic testing to screen patients with hereditary breast and ovarian cancer for known variants of BRCA1 and BRCA2 that exist in the Polish population (Janavicius, 2010). They demonstrate that the use of a simple genetic test focusing on five founder pathogenic variants can be a cost-effective first-line approach to identifying patients with common mutations in these genes. These patients may benefit from a rapid diagnosis and be treated with targeted therapies, such as PARP inhibitors. Whereas this work focused on known pathogenic mutations, Khanakji and Mifsud et al. address the issue that the pathogenicity of many BRCA2 mutations in patients remains unclear. By developing a gene-specific machine-learning model, the authors were able to predict the pathogenicity of BRCA2 mutations with high accuracy, as demonstrated by comparing their predictions with previously published functional data (Richardson et al., 2021). This tool may therefore aid in the classification of BRCA2 patient mutations, which may help to guide therapeutic approaches.

Rather than focus initially on the function and regulation of specific proteins, Huo et al. instead study the differential expression of more than 200 DNA repair genes in patients with hepatocellular carcinoma, with the intention of identifying expression signatures of prognostic value. By doing so, the authors determine a five-gene expression signature that could be used as an independent prognostic indicator. They suggest that measuring the deregulated expression of these genes may therefore be useful in clinical decision-making for patients with liver cancer. In the article by Yang et al. the authors also focus on identifying a signature of genome stability deregulation that could be used prognostically. Uniquely for this Research Topic, however, the authors focus not on proteins or other signaling molecules, but on the aberrant expression of long non-coding RNAs (lncRNAs) that regulate genome instability-related processes (Guo et al., 2021). Their work implies that evaluating a subset of these lncRNAs might be useful as a proxy measurement for a deficient DNA damage response and be predictive of drug resistance and survival in patients with non-small cell lung cancer.

The publications in this Research Topic thereby provide insight into the essential roles that regulatory networks play in the genome stability pathways, and how their misregulation may be used for cancer diagnostics and treatment.
